# Brazilian lung transplantation: an expanding universe

**DOI:** 10.1590/S1516-31802009000600001

**Published:** 2010-05-21

**Authors:** Paulo Manuel Pêgo-Fernandes, Alessandro Wasum Mariani

**Affiliations:** I MD, PhD. Associate professor, Department of Cardiopneumonology, Faculdade de Medicina da Universidade de São Paulo (FMUSP), São Paulo, Brazil. E-mail: paulo.fernandes@incor.usp.br; II MD. Surgeon in the Lung Transplant Group, Instituto do Coração (InCor), Hospital das Clínicas (HC), Faculdade de Medicina da Universidade de São Paulo (FMUSP), São Paulo, Brazil. E-mail: alessandro_mariani@hotmail.com

After the Toronto Lung Transplant Group succeeded in performing the first human lung transplant with a reasonable outcome in 1983,^1^ several groups around the world started to show great interest in this complex procedure. Since then, this operation has become an accepted treatment for end-stage lung disease. Data from the Registry of the International Society for Heart and Lung Transplantation (ISHLT) published in the 2008 ISHLT Report show the rapid increase in use of the procedure worldwide. Over the first ten years (1985-1995), the number of lung transplantations performed per year went from 14 to 1366. The second decade of the registry (1996-2006) demonstrates that a plateau of approximately 1700 to 2200 procedures per year was reached.^2^

The major factor limiting further increases in the number of lung transplantations is the shortage of donor lungs.^3^ Even in the most active centers, the median number of lungs harvested represents only 15% of all cadaver donors, whereas kidneys and livers are harvested from 88% and hearts from 30% of deceased donors. These disparities are probably due to the vulnerability of lungs to potential complications that often arise before donor brain death, such as thoracic trauma or aspiration, and to other complications arising afterwards, such as ventilator-associated lung injury, pneumonia or neurogenic pulmonary edema.^4^

In Brazil, the first lung transplantation was performed in 1989 at Santa Casa de Misericórdia de Porto Alegre. Soon after this, in 1990, the first patient in the state of São Paulo was operated at Hospital São Paulo, Universidade Federal de São Paulo (Unifesp). Within the same year, another patient received a transplant at Instituto do Coração (InCor), Hospital das Clínicas (HC), Faculdade de Medicina da Universidade de São Paulo (FMUSP). The 1990s was a decade marked by the emergence of several groups, but after those first years, many of them shut down their programs or limited their experience to a few cases. The reasons for this probably include not only irregular outcomes but also economic factors, since the maintenance of a lung transplant program is very expensive. Data from the Brazilian Transplant Registry indicate that in 1998, only 10 lung transplantations were performed in Brazil. The subsequent years showed a slow but positive change in this situation: in 2001, 23 lung transplants were performed in Brazil; in 2004, 46 cases; and in 2008, 53 cases.

The considerable improvements in overall outcome are clearly demonstrated by the 2008 ISHLT Report.^2^ Comparing different groups of years, the period from 1988 to 1994 had an estimated half-life of 3.9 years; 1995 to 1999, 4.5 years; and 2000 to 2006, 5.5 years.

Improved outcomes have also been seen in Brazil. In 2008, the Heart Institute (InCor) Lung Transplant Group calculated a Kaplan-Meier actuarial curve from the 85 cases included in our current series (2003-2008), which indicated that the likelihood of five-year survival was 63.15%.^5^ This result is clearly in accordance with the ISHLT data.^2^

If the numbers of lung transplantations have increased over the past 10 years, the number of groups performing the procedure has not dramatically changed. Nationwide in Brazil, there are 11 registered lung transplantation teams, although only three of them currently show signs of activity in 2009: Hospital das Clínicas, Universidade Federal de Minas Gerais (UFMG); Santa Casa de Misericórdia de Porto Alegre; and InCor HC-FMUSP.^6^ On the other hand, many groups and hospitals in different states have shown interest in starting or even restarting lung transplantation programs. The main marker for this tendency is the growing search for lung transplantation training among thoracic surgeons and pneumonologists. Regular training programs and short courses, for example on lung harvest capacitation, are being creating in order to provide professionals with appropriate skills.

Another marker of the growing interest in lung transplantations in Brazil is the increasing medical research on this subject. The number of scientific papers published by Brazilian lung transplantation groups has been increasing, and the subject of lung transplantation has frequently been discussed at medical congresses relating to associated specialties. For example, the InCor Lung Transplant Group is conducting a major study on donor lung reconditioning, based on ex-vivo lung evaluation. In some years’ time, this may have the potential to dramatically increase the lung harvest percentage, consequently leading to a major increase in the number of lung transplantations.

Lung transplantation in Brazil today is experiencing a golden age, with increasing numbers of transplantations year by year and growth in the numbers of interested groups and hospitals planning to establish regular programs. This situation is being propelled forward by the good outcomes shown over the last few years, and also by the growing interest in the development of organ and tissue transplantation demonstrated by the government and by society.
